# Obesity epidemic in urban Tanzania: a public health calamity in an already overwhelmed and fragmented health system

**DOI:** 10.1186/s12902-020-00631-3

**Published:** 2020-09-29

**Authors:** Pedro Pallangyo, Zabella S. Mkojera, Naairah R. Hemed, Happiness J. Swai, Nsajigwa Misidai, Lucy Mgopa, Smita Bhalia, Jalack Millinga, Theophil L. Mushi, Lucia Kabeya, Aisha Omar, Alice Kaijage, Rydiness Mulashani, Silvia Mosha, Faustina Mwapinga, Mohamed Janabi

**Affiliations:** 1 Unit of Research, Jakaya Kikwete Cardiac Institute, P.O Box 65141, Dar es Salaam, Tanzania; 2 Cardiology, Jakaya Kikwete Cardiac Institute, P.O Box 65141, Dar es Salaam, Tanzania; 3 Nursing, Jakaya Kikwete Cardiac Institute, P.O Box 65141, Dar es Salaam, Tanzania

**Keywords:** Excess body weight, Over nutrition, Overweight, Obesity, Malnutrition, Developing world, Sub Saharan Africa, Dar Es Salaam, Urban Tanzania

## Abstract

**Background:**

Worldwide, the epidemiological and demographic transitions have resulted in nutrition shift characterized by an increased consumption of high energy fast food products. In just over 3 decades, overweight and obesity rates have nearly tripled to currently affecting over a third of the global population. Notwithstanding the ever present under-nutrition burden, sub Saharan Africa (SSA) is witnessing a drastic escalation of overweight and obesity. We aimed to explore the prevalence and associated factors for obesity among residents of Dar es Salaam city in Tanzania.

**Methods:**

Participants from this study were recruited in a community screening conducted during the Dar es Salaam International Trade Fair. Sociodemographic and clinical data were gathered using a structured questionnaire during enrollment. Dietary habits and anthropometric measurements were assessed using standard methods. All statistical analyses utilized STATA v11.0 software. Pearson Chi square and Student’s T-test were used to compare categorical and continuous variables respectively. Logistic regression analyses were used to assess for factors associated with BMI ≥ 25. All tests were 2-sided and *p* < 0.05 was used to denote a statistical significance.

**Results:**

A total of 6691 participants were enrolled. The mean age was 43.1 years and males constituted 54.2% of all participants. Over two-thirds of participants were alcohol consumers and 6.9% had a positive smoking history. 88.3% of participants were physically inactive, 4.7% had a history of diabetes mellitus and 18.1% were known to have elevated blood pressure. Overweight and obesity were observed in 34.8 and 32.4% of participants respectively. Among overweight and obese participants, 32.8% had a misperception of having a healthy weight. Age ≥ 40, female gender, a current working status, habitual breakfast skipping, poor water intake, high soft drink consumption, regular fast food intake, low vegetable and fruit consumption, alcohol consumption and hypertension were found to be independent associated factors for obesity.

**Conclusion:**

Amidst the ever present undernutrition in SSA, a significant proportion of participants had excess body weight. Concomitantly, the rates of physical inactivity and unhealthy eating are disproportionately high in Dar es Salaam. In view of this, community-based and multilevel public health strategies to promote and maintain healthy eating and physical activity require an urgent step-up in urban Tanzania.

## Background

Globally, the obesity epidemic is soaring and presage a serious public health threat. In just over 3 decades, overweight and obesity burden has nearly tripled to currently affecting over a third of the global population [1]. With the current pace, obesity is projected to affect more than half of the world population by 2030 [2, 3]. Obesity is associated with elevated risk of literally every chronic condition. For instance, 14% of heart failure, 23% of ischemic heart disease, 44% of diabetes, 70% of hypertension and up to 41% of cancer burden is attributable to excess body weight [4–6]. Furthermore, obesity carries an increased individual all-cause mortality risk and claims about 4 million lives annually [7, 8]. As the obesity pandemic escalates, its health consequences are inevitably accompanied by an overwhelming economic threat. At present, the global economic impact of obesity amounts to $2 trillion annually (i.e. 2.8% of global GDP) making it among the top three most costly manmade burdens of all times [2, 9].

Similar to many other low and middle income countries (LMICs), sub Saharan Africa (SSA) is witnessing a rapid transition from a predominantly underweight population to an overweight one [10, 11]. Currently, about 35% of SSA inhabitants have excess body weight and the World Health Organization estimates an up-to 50% prevalence in the urban areas [12–14]. Community studies from urban Tanzania have revealed escalating overweight and obesity rates between 24.1–34.5% and 19.2–23.4% respectively [15–18]. Owing to the enormous heterogeneity and unique genetic diversity within SSA region, it is pivotal to comprehensively study and quantify the burden and resultant effects of obesity for formation of feasible, effective and cost-effective strategies to address this pandemic before it becomes insurmountable. We aimed to explore the prevalence and associated factors for obesity among residents of Dar es Salaam city in Tanzania.

## Methods

### Study design, recruitment process, and definition of terms

This cross sectional community-based study was conducted in Dar es Salaam City between July 2018 and August 2019. Participants of this study were consecutively enrolled during the screening conducted by Jakaya Kikwete Cardiac Institute (JKCI) during the Dar es Salaam International Trade Fair. Participants came from all over Dar es Salaam to the trade fair grounds for various shopping activities. Moreover, participants didn’t have prior information regarding the screening activity and thus their inclusion was not only voluntary but also random. As per the 2018 statistics, the city of Dar es Salaam had a population of 6,048,000 [19]. For the purpose of this screening, we aimed to recruit at least 0.1% of the population i.e. ≥6048 persons.

Sociodemographic and clinical data were gathered during enrollment using a structured questionnaire developed for this study [Additional file 1] We also assessed perception of one’s body weight (prior anthropometric measurements) by asking them in what BMI range do they see themselves in. Height and weight measurements were taken from consented participants to calculate body mass index (BMI). Underweight, normal, overweight and obese categorization followed the standard BMI cut-off values i.e. BMI < 18.5, 18.5–24.9, 25–29.9, and ≥ 30.0 kg/m^2^ respectively [20]. Physical activity was assessed using the Physical Activity Vital Sign (PAVS) [21] whereby reported moderate-vigorous physical activity of 0 min/week, < 150 min/week, or ≥ 150 min/week was used to categorize participants as inactive, underactive or active respectively. Hypertension was defined as systolic blood pressure (SBP) > 140 mmHg and/or diastolic blood pressure (DBP) > 90 mmHg or use of blood pressure medications [22]. Several dietary habits were assessed during this study including, breakfast consumption, water intake, soft drinks intake, fast food consumption, vegetables & fruits intake, and alcohol drinking. For the purpose of this study we regarded breakfast taking in ≥5 days/week, water intake of ≥2 L/day, soft drink consumption in ≤2 days/week, fast food intake of ≤2 days/week and a > 4 days/week fruit and vegetable consumption as healthy cut-offs. Health insurance possession and health-seeking behavior assessment was based of self-report. Aside screening, JKCI staff also provided health education pertaining to NCDs and nutrition to all participants.

### Statistical analysis

All statistical analyses utilized STATA v11.0 software. Pearson Chi square and Student’s T-test were used to compare categorical and continuous variables respectively. Logistic regression analyses were used to assess for factors associated with BMI ≥ 25. Stepwise and forward selection procedures were used to add and assess the statistically significant variables in the multivariate regression model. The regression models were fitted with baseline covariates associated with overweight/obesity by bivariate analysis at the < 0.05 significance level. All tests were 2-sided and *p* < 0.05 was used to denote a statistical significance.

## Results

### Study population

A total of 6691 participants were enrolled in this study. Table 1 displays their sociodemographic and clinical characteristics. The mean age was 43.1 years and males constituted 54.2% of all participants. About 72% of participants had at least secondary school education and 82.4% had a regular income generating activity. Approximately 25% of all participants had a health insurance and 25.9% had a health check-up at least once in their life time. Over two-thirds of participants were regular alcohol consumers and 6.9% had a positive smoking history. Less than 12% of participants were physically active and 4.7% had a history of diabetes mellitus.

**Table 1:** Sociodemographic and Clinical Characteristics (*N* = 6691)

Characteristic	Frequency
**Age**	
Mean (SD)	43.1 (14.2)
Range	18–95
Age Group	
18–33 years	1978 (29.6%)
34–49 years	2540 (38.0%)
50–65 years	1699 (25.4%)
> 65	474 (07.0%)
**Sex**	
Male	3625 (54.2%)
Female	3066 (45.8%)
**Education**	
No Formal	205 (3.1%)
Primary	1692 (25.3%)
Secondary	2155 (32.2%)
University	2639 (39.4%)
**Occupation**	
Jobless	612 (9.1%)
Student	285 (4.3%)
Self Employed	3605 (53.9%)
Employed	1907 (28.5%)
Retired	282 (4.2%)
**Health Insurance**	
Yes	1678 (25.1%)
No	5013 (74.9%)
**Medical check-up** (lifetime)	
Yes	1733 (25.9%)
No	4958 (74.1%)
**Alcohol intake**	
Yes	4447 (66.5%)
No	2244 (33.5%)
**Smoking status**	
Never	6229 (93.1%)
Past	184 (2.8%)
Current	278 (4.1%)
**Physical Activity**	
Inactive	3892 (58.2%)
Underactive	2016 (30.1%)
Active	783 (11.7%)
**Body Mass Index**	
Mean	27.9 (5.8)
BMI Categories	
Underweight (BMI < 18.5 kg/m^2^)	181 (2.7%)
Normal (BMI 18.5–24.9 kg/m^2^)	2014 (30.1%)
Overweight (BMI 25–29.9 kg/m^2^)	2327 (34.8%)
Obese (BMI ≥ 30 kg/m^2^)	2169 (32.4%)
Morbid obesity (BMI ≥ 40 kg/m^2^)	203 (09.4%)
**Chronic Illness History**	
Hypertension	1212 (18.1%)
Diabetes	311 (4.7%)
**Blood Pressure**	
Negative Hypertension History	
Normotension	1595 (29.1%)
Pre-hypertension	2086 (38.1%)
Hypertension	1798 (32.8%)
Positive Hypertension History	
Awareness	1212 (40.3%)
Treatment	387 (31.9%)
Control	254 (21.0%)

### Weight perception, unhealthy habits and excess body weight

The mean BMI was 27.9 kg/m^2^. Overweight and Obesity were observed in 34.8 and 32.4% of participants respectively. Among overweight and obese participants, 67.2% had a perception of having excess body weight. Table 2 displays a comparison between BMI < 25 and BMI ≥ 25 on various sociodemographic and clinical characteristics. Participants with a BMI ≥ 25 were significantly older compared to their counterparts with BMI < 25 (45.4 vs 38.3, *p* < 0.001). Apart from the age group 18–33 years which had predominantly BMI < 25, all other age groups had at least twice as much overweight and obese individuals compared to normal weight individuals. Females consistently displayed higher rates of overweight and obesity compared to males (72.0% vs 63.1%, *p* < 0.001) across all age groups. Participants with ≤ primary education and those with regular income generating activity displayed higher rates of excess body weight (70.9% vs 65.7%, *p* < 0.001 and 68.9% vs 59.1%, *p* < 0.001 respectively). Regarding dietary habits, participants with BMI ≥ 25 displayed higher frequencies of skipping breakfast, less water intake, more soft drinks consumption, frequent fast food intake, less vegetable and fruit consumption and more alcohol intake compared to participants with normal body weight.

**Table 2:** Comparison between BMI < 25 & BMI ≥ 25 (*N* = 6691)

Characteristic	BMI < 25 (*n* = 2195)	BMI ≥ 25 (*n* = 4496)	***p***-value
**Age** (mean, SD)	38.3 (15.3)	45.4 (13.1)	**< 0.001**
**Age groups**			
18–33^a^	1074 (54.3%)	904 (45.7%)	–
34–49	620 (24.4%)	1920 (75.6%)	**< 0.001**
50–65	347 (20.4%)	1352 (79.6%)	**< 0.001**
> 65	154 (32.5%)	320 (67.5%)	**< 0.001**
**Sex**			
Female	858 (28.0%)	2208 (72.0%)	
Male	1337 (36.9%)	2288 (63.1%)	**< 0.001**
**Education level**			
≤ primary education	552 (29.1%)	1345 (70.9%)	
≥ secondary education	1643 (34.3%)	3151 (65.7%)	**< 0.001**
**Employment status**			
Currently working	1713 (31.1%)	3799 (68.9%)	
Not working (students, jobless, retired)	482 (40.9%)	697 (59.1%)	**< 0.001**
**Dietary habits**			
- Breakfast intake (days/week)	3.7	4.2	**< 0.001**
- Water intake (liters/day)	2.7	1.4	**< 0.001**
- Soft drinks consumption (days/week)	1.9	4.7	**< 0.001**
- Fast food consumption (days/week)	2.3	4.2	**< 0.001**
- Vegetables & Fruits intake (days/week)	4.6	2.3	**< 0.001**
- Alcohol drinkers	617 (28.1%)	1627 (36.2%)	**< 0.001**
**Smoking History**			
Ever smokers	172 (37.2%)	290 (62.8%)	
Never smokers	2023 (32.5%)	4206 (67.5%)	**0.04**
**Physical Activity**			
Inactive/Underactive	1906 (32.3%)	4002 (67.7%)	
Active	289 (36.9%)	494 (63.1%)	**0.01**
**Hypertension history**			
Positive	243 (20.0%)	969 (80.0%)	
Negative	1952 (35.6%)	3527 (64.4%)	**< 0.001**
**Elevated Blood Pressure**			
Normal	1573 (39.4%)	2419 (60.6%)	
Elevated	622 (23.0%)	2077 (77.0%)	**< 0.001**
**Perception about current body weight**			
(% perceiving themselves as overweight/obese)	116 (5.3%)	3022 (67.2%)	**< 0.001**

^a^reference group

A total of 1212 (18.1%) participants had a positive history of hypertension. Of these, 387 (31.9%) were on regular use of anti-hypertensive medications and 254 (21.0%) had attained a blood pressure control (i.e. BP < 140/90 mmHg). A total of 1798 (32.8%) were newly diagnosed with hypertension during this screening. Participants with excess body weight displayed a higher likelihood of having both an elevated blood pressure (25.3% vs 16.0%, *p* < 0.001) and poor blood pressure control (16.4% vs 7.6%, *p* < 0.001) compared to their counterparts with normal weight.

Table 3 display the results of logistic regression analyses for factors associated with excessive body weight. During bivariate analysis, all 13 characteristics that were included in our regression model showed a significant association with excess body weight. We then ran multivariate logistic regression analyses during which 10 out of the 13 factors proved to be independently associated with excessive body weight. These included: age ≥ 40 (OR 2.3, 95% CI 1.8–3.0, *p*-value < 0.001), female sex (OR 1.8, 95% CI 1.4–2.3, *p*-value < 0.001), a current working status (OR 1.8, 95% CI 1.4–2.5, *p*-value < 0.001), habitual breakfast skipping (OR 1.6, 95% CI 1.3–2.1, *p*-value < 0.001), poor water intake (OR 24.2, 95% CI 18.4–31.7, *p*-value < 0.001), high soft drink consumption (OR 75.1, 95% CI 57.6–98.0, *p*-value < 0.001), regular fast food intake (OR 11.2, 95% CI 8.8–14.4, *p*-value < 0.001), low vegetable and fruit consumption (OR 10.1, 95% CI 7.7–13.3, *p*-value < 0.001), alcohol consumption (OR 1.7, 95% CI 1.3–2.2, *p*-value < 0.001) and elevated blood pressure (OR 1.3, 95% CI 1.0–1.7, *p*-value < 0.05).

**Table 3:** Associated Factors for Excessive Body Weight

Characteristic	Comparative	OR	95% CI	***p***-value	Adj.OR	95% CI	***p***-value
Age > =40	Age < 40	3.0	2.7–3.3	< 0.001	2.3	1.8–3.0	**< 0.001**
Female	Male	1.5	1.4–1.7	< 0.001	1.8	1.4–2.3	**< 0.001**
≤ Primary Education	≥ Secondary Education	1.3	1.1–1.4	< 0.001	1.2	0.9–1.5	0.27
Currently Working	Unemployed	1.5	1.3–1.7	< 0.001	1.8	1.4–2.5	**< 0.001**
Inactive	Active	1.2	1.1–1.4	< 0.01	1.4	0.97–2.0	0.07
Often skips breakfast (≥ 4 days/week)	Regular takers (< 4 days)	1.7	1.5–1.9	< 0.001	1.6	1.3–2.1	**< 0.001**
Water intake < 2 L/day	Intake ≥2 L/day	22.4	19.2–26.1	< 0.001	24.2	18.4–31.7	**< 0.001**
Soft drinks intake > 2 days /week	≤2 days/week	67.0	57.0–78.7	< 0.001	75.1	57.6–98.0	**< 0.001**
Fast food intake > 2 days/week	≤2 days/week	9.9	8.8–11.1	< 0.001	11.2	8.8–14.4	**< 0.001**
Vegetables & Fruits intake ≤4 days/week	> 4 days/week	11.9	10.4–13.7	< 0.001	10.1	7.7–13.3	**< 0.001**
Positive alcohol history	Non-drinkers	1.5	1.3–1.6	< 0.001	1.7	1.3–2.2	**< 0.001**
Positive history of Hypertension	Negative history	2.2	1.9–2.6	< 0.001	1.2	0.9–1.7	0.30
Elevated BP	Normal BP	2.2	1.9–2.4	< 0.001	1.3	1.0–1.7	**< 0.05**

## Discussion

Worldwide, the epidemiological and demographic transitions have resulted in nutrition shift characterized by an increased consumption of sugar-sweetened beverages and energy-dense fast food products [1–4, 8, 11, 13, 15]. Consequently, the world is now heavier than ever before in the existence of mankind. The drivers of obesity are complex and multifactorial and poses a substantial clinical challenge in both its prevention and management. With over 2 billion people affected worldwide, obesity is attributed to an array of adverse health-related outcomes including increased morbidity, decreased productivity, impaired quality of life, excessive healthcare utilization and increased all-cause mortality risk [2, 4–9]. Notwithstanding the ever present under-nutrition burden, sub Saharan Africa is witnessing a drastic escalation of overweight and obesity [10–18].

In this present study, the mean BMI of study participants fell in the overweight range (i.e. 27.9 kg/m^2^) and about one third were found to be obese. These rates are substantially higher compared to findings from studies conducted in the same region in 1996, 2008 and 2012 which revealed obesity rates of 12.6% [23], 19.2% [16] and 23.4% [17] respectively. Simultaneously, overweight/obesity rates among children is apparently rising at an alarming pace in Dar es Salaam [24–27]. While a large majority (94.7%) of individuals with normal weight had an accurate perception regarding their weight, approximately a third of individuals with excess body weight had a misperception that they have a healthy weight. In unison to our findings, earlier studies [17, 28] conducted in this region revealed similar rates of weight misperception among participants with excess body weight. Nonetheless, despite of the existing controversy regarding how the perception of being overweight relates to weight related lifestyle behaviors; right perception on one’s body image is likely to have a positive influence on health behaviors [29–31]. Conversely, cultural and social influences particularly in SSA have been shown to impact body image perception [32]. For instance in some communities where excess body weight is perceived as a sign of beauty, health, fertility, power and/or financial prosperity, exacerbation of eating disorders is observed [33–36].

Similar to previous studies in many other settings, obesity was found to increase exponentially with increasing age [15–18, 23–28]. Moreover, across all age groups (except for 18–33 years), we observed a disproportionate predominance of overweight and obese individuals. Ominously, the preponderance of obesity across most age groups is a warning sign that without urgent intervention, we are likely to witness an overweight/obesity domination across all population subgroups. Additionally, an increasing body of knowledge is suggesting that obesity may speed up the aging process by inducing senescence [37–39]. Women displayed an 80% increased likelihood of having excess body weight compared to men in this present study. In contrast to high income countries, women living in lower and middle income countries bear a disproportionate burden of obesity [11, 15–18, 23–28]. Several factors including sociocultural dynamics, physical activity patterns, pregnancy, parity and menopause have been implicated in the body weight disparities between women and men residing in the developing world [40, 41].

Owing to urbanization, residents of the developing world are exposed to less energy-demanding jobs, increased sedentary lifestyles, and adoption of a western diet which are convincingly implicated as causative factors in accelerating the obesity epidemic [10–18, 42, 43]. Physical inactivity is among the primary contributors to the obesity epidemic all over the globe. Nearly 90% of participants in this study were categorized as being inactive or underactive. Contrary to our findings, earlier local studies revealed physical activity rates between 83.2% [16] to 92.6% [44]. Such discrepancy could partly be explained by the variation in the assessment tools used to assess for physical activity among studies, however, based on the observed obesity rates such rates are likely to be a true reflection. Although we observed a negative association between smoking and obesity, alcohol consumption in this study was associated with a 70% increased likelihood of having excess body weight. Correspondingly, numerous studies all over the globe have revealed higher obesity rates among alcohol drinkers compared to non-drinkers [45–47].

Several unhealthy eating habits including skipping breakfast, poor water intake, high consumption of soft drinks and fast foods, and low intake of vegetables and fruits were observed in this present study. Skipping breakfast has been shown to increase one’s risk of becoming obese in various studies [48–51]. Participants who skipped breakfast for at least 4 days in a week displayed a 60% increased likelihood of being obese in this present study. Adequate hydration is known to reduce overeating and increase lipolysis thus promoting a healthy weight [52]. Despite of a low evidence for a causal association, numerous longitudinal studies have demonstrated a significant association between inadequate hydration and elevated BMI/obesity [53, 54]. In this current study, participants who reported an average water intake of ≤2 L/day had a 24 times odds of being overweight or obese compared to their counterparts who consumes more than 2 L a day.

Globally, the consumption of sugar-sweetened beverages has increased in recent times. Participants who consumed beverages for ≥3 days/week displayed a 75 times increased likelihood of having excess body weight compared to those who drinks in 2 days or less. Studies from different parts of the globe have also demonstrated how sweetened beverages is accelerating obesity rates in various communities [55–57]. Likewise, mushrooming of franchised fast food particularly in the developing world is concomitantly fuelling the obesity epidemic [58–61]. Participants who consume fast food for 3 or more days in a week displayed an 11 times chance of being overweight or obese compared to their counterparts who seldom consumes fast food. Currently in Dar es Salaam the price of soda is similar to that of bottled drinking water while on average fast food is cheaper than a healthy locally-made meal. Several previous studies have also demonstrated an inverse association between fruit/vegetable consumption and body adiposity [62–66]. Study participants with low fruit/vegetable consumption displayed a 10 times odds of having excess body weight compared to regular fruit/vegetables consumers. Overall, consistent with earlier studies on this topic, similar unhealthy habits have been associated with increasing obesity rates in this African population. More worryingly though is the rapid transformation, escalation and adoption of the unhealthy lifestyle habits observed in this present study compared to studies conducted in the same setting some years ago.

### Strengths and limitations

The strengths of our study include a sufficiently large sample to estimate the prevalence of obesity and to explore subgroup (i.e. sex and age-specific) burdens with adequate precision. Furthermore, we used rigorous and standardized protocols for data collection and utilized qualified and competent personnel in all measurements. Despite our efforts, this study is not short of limitations. The cross-sectional nature of this study dictated our risk-factor assessment particularly dietary habits and physical activity to rely on self-reported measures thus recall bias and reverse causation cannot be precluded. Moreover, BMI was the only measure of adiposity utilized in this study. Nevertheless, it remains the most frequently used measure in epidemiologic studies. Additionally, owing to the less comprehensive dietary intake data, our association results should be interpreted with some caution. Furthermore, the nature of the study area inevitably limited participation of some population groups (i.e. children and elderly) and thus a sampling bias is probable. Lastly, single-occasion BP measurements (as taken in this study) has the potential to overestimate the hypertension burden.

## Conclusions

The burden of obesity in urban Tanzania has reached the boiling point. Parallel to this, reciprocal trajectories of Non Communicable Diseases are also expected to intensify if measures to curb the obesity epidemic are not taken timely and aggressively. These findings have imperative public health ramifications and call for urgent and collaborative efforts from healthcare workers, government agencies and policy makers to combat the escalating obesity crisis before it becomes unabated. Community-based and multilevel public health strategies to promote and maintain healthy diet and physical activity including price reduction for healthy foods, increasing the price of unhealthy foods, incorporating nutrition health education and physical activity programs in schools and workplace, and initiation of social support interventions for physical activity (i.e. walking groups, jogging clubs, and exercise buddy system) require an urgent step-up in urban Tanzania.

## Supplementary information

**Additional file 1.**

## Data Availability

The datasets used and/or analysed during the current study available from the corresponding author on reasonable request.

## Abbreviations

95% CI: 95% Confidence Interval
BMI: Body mass index
BP: Blood pressure
DBP: Diastolic blood pressure
GDP: Gross Domestic Product
JKCI: Jakaya Kikwete Cardiac Institute
LMIC: Low and middle income counties
NCDs: Non-communicable diseases
OR: Odd Ratio
PAVS: Physical Activity Vital Sign
SSA: Sub Saharan Africa
SBP: Systolic blood pressure
WHO: World Health Organization

## References

[1] Hruby A, Hu FB (2015). The epidemiology of obesity: a big picture. Pharmacoeconomics.

[2] Dobbs R, Sawers C, Thompson F (2014). Overcoming Obesity: An Initial Economic Analysis.

[3] Kelly T, Yang W, Chen CS (2008). Global burden of obesity in 2005 and projections to 2030. Int J Obes.

[4] World Health Organization (2013). Obesity and overweight, World Health Organization Fact Sheet.

[5] Must A, Spadano J, Coakley EH (1999). The disease burden associated with overweight and obesity. JAMA.

[6] Kenchaiah S, Evans JC, Levy D (2002). Obesity and the risk of heart failure. N Engl J Med.

[7] Ramsay M, Crowther NJ, Agongo G (2018). Regional and sex-specific variation in BMI distribution in four sub-Saharan African countries: The H3Africa AWI-Gen study. Glob Health Action.

[8] The GBD (2017). 2015 obesity collaborators. Health effects of overweight and obesity in 195 countries over 25 years. N Engl J Med.

[9] Tremmel M, Gerdtham U, Nilsson PM, Saha S (2017). Economic burden of obesity: a systematic literature review. Int J Environ Res Public Health.

[10] Lim SS, Vos T, Flaxman AD, Danaei G, Shibuya K (2012). A comparative risk assessment of burden of disease and injury attributable to 67 risk factors and risk factor clusters in 21 regions, 1990–2010: a systematic analysis for the global burden of disease study 2010. Lancet.

[11] Ford ND, Patel SA, Narayan KM (2017). Obesity in low- and middle-income countries: burden, drivers, and emerging challenges. Annu Rev Public Health.

[12] WHO (2010). Global status report on non-communicable diseases.

[13] Abrahams Z, Mchiza Z, Steyn NP (2011). Diet and mortality rates in sub-Saharan Africa: stages in the nutrition transition. BMC Public Health.

[14] Sodjinou R, Agueh V, Fayomi B, Delisle H (2008). Obesity and cardiometabolic risk factors in urban adults of Benin: relationship with socio-economic status, urbanisation, and lifestyle patterns. BMC Public Health.

[15] Ajayi IO, Adebamowo C, Adami H (2016). Urban–rural and geographic differences in overweight and obesity in four sub-Saharan African adult populations: a multi-country cross-sectional study. BMC Public Health.

[16] Shayo GA, Mugusi FM (2011). Prevalence of obesity and associated risk factors among adults in Kinondoni municipal district, Dar es Salaam Tanzania. BMC Public Health.

[17] Muhihi AJ, Njelekela MA, Mpembeni R (2012). Obesity, overweight, and perceptions about body weight among middle-aged adults in Dar Es Salaam, Tanzania. ISRN Obes.

[18] Njelekela M, Kuga S, Nara Y (2002). Prevalence of obesity and dyslipidemia in middle-aged men and women in Tanzania, Africa: relationship with resting energy expenditure and dietary factors. J Nutr Sci Vitaminol (Tokyo).

[19] MacroTrends. Dar es Salaam, Tanzania Population 1950–2020. https://www.macrotrends.net/cities/22894/dar-es-salaam/population.

[20] Weir CB, Jan A (2019). BMI classification percentile and cut off points. StatPearls.

[21] Golightly YM, Allen KD, Ambrose KR (2017). Physical activity as a vital sign: a systematic review. Physical Activity as a Vital Sign: A Systematic Review. Prev Chronic Dis.

[22] Chobanian AV, Bakris GL, Black HR (2003). Seventh report of the joint national committee on prevention, detection, evaluation, and treatment of high blood pressure. Hypertension.

[23] Edwards R, Unwin N, Mugusi F (2000). Hypertension prevalence and care in an urban and rural area of Tanzania. J Hypertens.

[24] Mpembeni RN, Muhihi AJ, Maghembe M (2014). Overweight, obesity and perceptions about body weight among primary schoolchildren in Dar Es Salaam, Tanzania. Tanzan J Health Res.

[25] Pangani IN, Kiplamai FK, Kamau JW, Onywera VO (2016). Prevalence of overweight and obesity among primary school children aged 8–13 years in Dar es Salaam City, Tanzania. Adv Prev Med.

[26] Mwaikambo SA, Leyna GH, Killewo J (2015). Why are primary school children overweight and obese? A cross sectional study undertaken in Kinondoni district, Dar-es-salaam. BMC Public Health.

[27] Mosha TCE, Fungo S (2010). Prevalence of overweight and obesity among children aged 6–12 years in Dodoma and Kinondoni municipalities, Tanzania. Tanzan J Health Res.

[28] Shekalaghe BA, Urassa DP. Body Mass Index and self perceptions of weight status among outpatients attending hospital health facilities in Dar es Salaam. 2015; 12(1).

[29] Sirirassamee T, Phoolsawat S, Limkhunthammo S (2018). Relationship between body weight perception and weight-related behaviours. J Int Med Res.

[30] Edwards NM, Pettingell S, Borowsky IW (2010). Where perception meets reality: self-perception of weight in overweight adolescents. Pediatr.

[31] Fredrickson J, Kremer P, Swinburn B (2015). Weight perception in overweight adolescents: associations with body change intentions, diet and physical activity. J Health Psychol.

[32] Gluck ME, Geliebter A (2002). Racial/ethnic differences in body image and eating behaviors. Eat Behav.

[33] Aryeetey RNO (2016). Perceptions and experiences of overweight among women in the Ga East District, Ghana. Front Nutr.

[34] Ulijaszek SJ, Lofink H (2006). Obesity in biocultural perspective. Annu Rev Anthropol.

[35] Madanat H, Hawks SR, Angeles HN (2011). Obesity and body size preferences of Jordanian women. Health Educ Behav.

[36] Ettarh R, Van de Vijver S, Oti S, Kyobutungi C (2013). Overweight, obesity, and perception of body image among slum residents in Nairobi, Kenya, 2008-2009. Prev Chronic Dis.

[37] Salvestrini V, Sell C, Lorenzini A (2019). Obesity May Accelerate the Aging Process. Front Endocrinol (Lausanne).

[38] Valdes AM, Andrew T, Gardner JP (2005). Obesity, cigarette smoking, and telomere length in women. Lancet.

[39] Nevalainen T, Kananen L, Marttila S (2017). Obesity accelerates epigenetic aging in middle-aged but not in elderly individuals. Clin Epigenetics.

[40] Case A, Menendez A (2009). Sex differences in obesity rates in poor countries: evidence from South Africa. Econ Hum Biol.

[41] Kanter R, Caballero B (2012). Global gender disparities in obesity: a review. Adv Nutr.

[42] Popkin BM (2006). Global nutrition dynamics: the world is shifting rapidly toward a diet linked with noncommunicable diseases. Am J Clin Nutr.

[43] Prentice AM (2006). The emerging epidemic of obesity in developing countries. Int J Epidemiol.

[44] Mashili FL, Kagaruki GB, Mbatia J (2018). Physical Activity and Associated Socioeconomic Determinants in Rural and Urban Tanzania: Results from the 2012 WHO-STEPS Survey. Int J Popul Res.

[45] Price AJ, Crampin AC, Amberbir A (2018). Prevalence of obesity, hypertension, and diabetes, and cascade of care in sub-Saharan Africa: a cross-sectional, population-based study in rural and urban Malawi. Lancet Diabetes Endocrinol.

[46] Traversy G, Chaput J (2015). Alcohol consumption and obesity: an update. Curr Obes Rep.

[47] Lourenço S, Oliveira A, Lopes C (2012). The effect of current and lifetime alcohol consumption on overall and central obesity. Eur J Clin Nutr.

[48] Watanabe Y, Saito I, Henmi I (2014). Skipping breakfast is correlated with obesity. J Rural Med.

[49] Okada C, Tabuchi T, Iso H (2018). Association between skipping breakfast in parents and children and childhood overweight/obesity among children: a nationwide 10.5-year prospective study in Japan. Int J Obes.

[50] Lazzeri G, Ahluwalia N, Niclasen B (2016). Trends from 2002 to 2010 in daily breakfast consumption and its socio-demographic correlates in adolescents across 31 countries participating in the HBSC study. PLoS One.

[51] Huang CJ, Hu HT, Fan YC (2010). Associations of breakfast skipping with obesity and health-related quality of life: evidence from a national survey in Taiwan. Int J Obes.

[52] Thornton SN (2016). Increased hydration can be associated with weight loss. Front Nutr.

[53] Chang T, Ravi N, Plegue MA (2016). Inadequate hydration, BMI, and obesity among US adults: NHANES 2009–2012. Ann Fam Med.

[54] Fetissov SO, Thornton SN (2009). Hypovolemia-induced obesity and diabetes. Metabolism..

[55] Malik VS, Schulze MB, Hu FB (2006). Intake of sugar-sweetened beverages and weight gain: a systematic review. Am J Clin Nutr.

[56] Lim L, Banwell C, Bain C (2014). Sugar sweetened beverages and weight gain over 4 years in a Thai national cohort--a prospective analysis. PLoS One.

[57] Ruanpeng D, Thongprayoon C, Cheungpasitporn W, Harindhanavudhi T (2017). Sugar and artificially sweetened beverages linked to obesity: a systematic review and meta-analysis. QJM..

[58] Al-Otaibi HH, Basuny AM (2015). Fast food consumption associated with obesity/overweight risk among university female student in Saudi Arabia. Pak J Nutr.

[59] Alfawaz HA (2012). The relationship between fast food consumption and BMI among university female students. Pak J Nutr.

[60] Daradkeh G, Al Muhannadi A, Chandra P, Al Hajr M. Fast Food vs Healthy Food Intake and Overweight/Obesity Prevalence among Adolescents in the State of Qatar. J Obes Treat Weight Manag. 2018;1(1):001.

[61] Fraser LK, Clarke GP, Cade JE, Edwards KL (2012). Fast food and obesity: a spatial analysis in a large United Kingdom population of children aged 13-15. Am J Prev Med.

[62] Nour M, Lutze SA, Grech A, Allman-Farinelli M (2018). The relationship between vegetable intake and weight outcomes: a systematic review of cohort studies. Nutrients..

[63] Yu ZM, DeClercq V, Cui Y (2018). Fruit and vegetable intake and body adiposity among populations in Eastern Canada: the Atlantic Partnership for Tomorrow’s Health Study. BMJ Open.

[64] Mytton OT, Nnoaham K, Eyles H, Scarborough P, Ni Mhurchu C (2014). Systematic review and meta-analysis of the effect of increased vegetable and fruit consumption on body weight and energy intake. BMC Public Health.

[65] Hebden L, O’Leary F, Rangan A, Singgih LE, Hirani V, Allman-Farinelli M (2017). Fruit consumption and adiposity status in adults: a systematic review of current evidence. Crit Rev Food Sci Nutr.

[66] Schwingshackl L, Hoffmann G, Kalle-Uhlmann T, Arregui M, Buijsse B, Boeing H. Fruit and vegetable consumption and changes in anthropometric variables in adult populations: a systematic review and meta-analysis of prospective cohort studies. PLoS One. 2015;10(10):e0140846. 10.1371/journal.pone.0140846 PMC4608571 26474158

