# Supplementary material for: Obesity epidemic in urban Tanzania: a public health calamity in an already overwhelmed and fragmented health system
Source: BMC Endocr Disord. 2020 Sep 29;20:147. doi: 10.1186/s12902-020-00631-3 (PMC7526153; doi:10.1186/s12902-020-00631-3)
Supplement: Supplementary file 1 — Additional file 1. [file 12902_2020_631_MOESM1_ESM.doc]

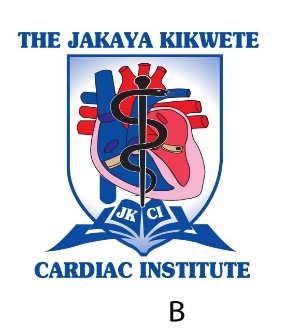
**Questionnaire.**

**S/N: ____________**

**Demographics & Medical history:**

1. **Age**: __________
2. **Sex**: Male Female
3. **Education**: No formal Primary Secondary University
4. **Occupation**: jobless student self-employed employed retired

1. Do you have a **health insurance?**: Yes No
2. Have you ever gone for a **health check-up** when you had no signs of illness? Yes No
3. Do you take **alcohol** ? yes no
4. Do you smoke **cigarette**? Never Past Current
5. On average, how many days in a week do you **exercise**? Days:_____ ; Duration/Day: ____hrs
6. What is your **perception** about your current **body weight**?

Underweight Normal Overweight

1. Do you have a history of high blood pressure (**hypertension**)? Yes No
2. Do you have a history of **diabetes mellitus**? Yes No

**Dietary assessment.**

1. On average, how much **water** do you consume in 24hours? ________ liters
2. On average, on how many days of the week do you take **breakfast**? _______ days
3. On average, on how many days of the week do you consume **soft drinks**? ______ days
4. On average, on how many days of the week do you consume **fast foods**? ______ days
5. On average, on how many days of the week do you consume **vegetables** or **fruits**? ____days

**Key measurements.**

**Weight:** _______ kg **; Height:** _________cm

**BP**: ___________ mmHg **PR**: _______ b/min

**Blood Sugar**: _____ mmol/L fasting random ;
